# Prioritized Detection of Personally Familiar Faces

**DOI:** 10.1371/journal.pone.0066620

**Published:** 2013-06-21

**Authors:** Maria Ida Gobbini, Jason D. Gors, Yaroslav O. Halchenko, Courtney Rogers, J. Swaroop Guntupalli, Howard Hughes, Carlo Cipolli

**Affiliations:** 1 Dipartimento di Medicina Specialistica, Diagnostica e Sperimentale, University of Bologna, Bologna, Italy; 2 Department of Psychological and Brain Sciences, Dartmouth College, Hanover, New Hampshire, United States of America; Cuban Neuroscience Center, Cuba

## Abstract

We investigated whether personally familiar faces are preferentially processed in conditions of reduced attentional resources and in the absence of conscious awareness. In the first experiment, we used Rapid Serial Visual Presentation (RSVP) to test the susceptibility of familiar faces and faces of strangers to the attentional blink. In the second experiment, we used continuous flash interocular suppression to render stimuli invisible and measured face detection time for personally familiar faces as compared to faces of strangers. In both experiments we found an advantage for detection of personally familiar faces as compared to faces of strangers. Our data suggest that the identity of faces is processed with reduced attentional resources and even in the absence of awareness. Our results show that this facilitated processing of familiar faces cannot be attributed to detection of low-level visual features and that a learned unique configuration of facial features can influence preconscious perceptual processing.

## Introduction

The capacity to detect ecologically relevant stimuli quickly has adaptive advantages. Previous work has demonstrated that stimuli that signal threat are processed preferentially in conditions of increased attentional load and without awareness [1], [2]. Perceiving socially relevant stimuli quickly, including those that can facilitate social exchanges in addition to those that signal threat, is essential for adaptive behavior. Faces convey important information for non-verbal communication. A bias toward faces is already present early in life [3]. Upright faces are detected preferentially, relative to inverted faces, even in the absence of conscious awareness [4], [5]. Signals expressed by faces that manifest interest, the desire to catch one’s attention, or the intention to engage in a social interaction such as eye gaze and head direction also are processed in the absence of conscious awareness [6], [7].

Face identity plays a central role in detection of in-group members and friends and plays a central role in the way we approach others. We wanted to investigate whether detection of personally familiar faces involves mechanisms that require fewer attentional resources than do mechanisms for detection of faces of strangers and whether these mechanisms operate even when faces are not consciously visible. We conducted two behavioral experiments on detection of personally familiar faces and faces of strangers while manipulating the attentional load and awareness of the stimuli.

In the first experiment we used a Rapid Serial Visual Presentation (RSVP) to measure detection rate for personally familiar faces as compared to faces of strangers during the attentional blink. The attentional blink is characterized by reduced detection of a second visual target (T2), embedded in a stream of rapidly presented visual stimuli, when it follows an initial target (T1) after an interval of 200–500 ms [8], [9]. Emotionally salient stimuli such as faces with fearful expression or emotionally meaningful words are less affected by the attentional blink than are neutral faces and words [10], [11]. Familiar faces represent a class of socially relevant stimuli. The capacity for quickly identifying friends not only has an important ecological value but also determines the way we approach and interact with others. For example, face categorization is faster when faces are personally familiar [12]. Better detection of one class of stimuli, as compared to another, during the attentional blink indicates that processing of the favored stimuli requires fewer attentional resources. Such processing facilitates orientation of attention to prioritized objects and events and extraction of information that may be important for adaptive behavior. Thus, better detection of familiar faces during the attentional blink would suggest learned processes that facilitate directing cognitive resources to in-group conspecifics.

In the second experiment we used continuous interocular flash suppression to render stimuli invisible. Interocular suppression with binocular rivalry is a well-established method to manipulate awareness [13], [14], [15]. Using this technique, we assessed whether personally familiar faces break through suppression faster than faces of strangers.

Our two experiments provide convergent results on prioritized detection of familiar faces. Consistent with our hypothesis, the results show that personally familiar faces, as compared to faces of strangers, are more readily detected during the attentional blink and break through interocular suppression faster. These findings suggest that personally familiar faces are recognized by processes that operate outside of the focus of attention and without visual awareness. Such enhanced detection may facilitate allocation of attention and awareness to these socially salient faces.

## Experiment 1 - Rapid Serial Visual Presentation

### Methods

#### Subjects

Thirteen healthy, right-handed volunteers with normal or corrected-to-normal vision and no record of neurological or psychiatric illness participated in the study (7 male, 6 female, mean age 26±7 year). Before the experiment, each participant signed an informed consent approved by the local ethical committee (IRB protocol 21200). Subjects were compensated for their participation.

#### Stimuli

**Figure 1:**
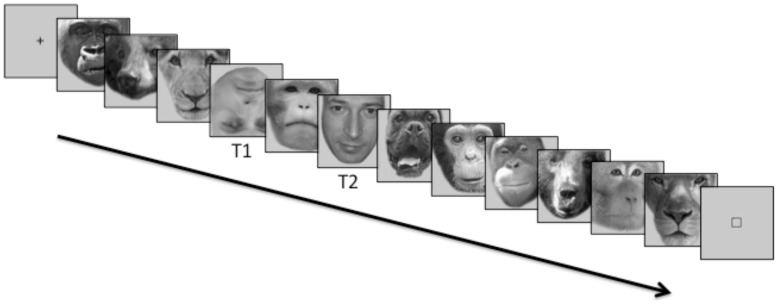
Attentional blink paradigm. Faces of different categories of mammals were used as distracters while human faces were presented as targets. The first target (T1) was an inverted face (always a face of a stranger different from those used as T2) and the second target (T2) was an upright face that was either a personally familiar face or the face of a stranger. Stimuli were presented for 80 ms with no interval between stimuli.

Faces of different categories of mammals such as bears, dogs, non-human primates, and lions were used as distracters (fillers). Human faces were presented as targets. The first target (T1) was an inverted face and the second target (T2) was an upright face that was either a personally familiar face or the face of a stranger (Figure 1). The inverted face (T1) was always a face of a stranger different from those used as T2. We chose inverted faces of strangers as T1 to make the nature of the task, human face detection, clear, and to make T1 and T2 clearly distinct. Inverted faces evoke strong responses in the face-selective occipital and fusiform cortices, suggesting that they use face-specific resources, even though they do not evoke a representation of individual identity that is as distinct [16]. Thus, inverted faces appear to engage face-specific cognitive resources.

Participants provided pictures of six personally familiar faces. Personally familiar individuals were chosen among relatives and long term friends with whom the participants reported to have a good relationship. Two different images of each individual were used, for a total of 12 familiar face images. Faces of strangers for each subject were chosen from the familiar faces for the other participants and were age and gender matched with that subject’s personally familiar faces. Faces were converted to grayscale, cropped to remove the hair, body parts, clothing, and background, then superimposed on a uniform gray background using Adobe Photoshop (San Jose, CA) to minimize pictorial memory for the stimuli. Images were normalized to have the same mean luminance and contrast. Subjects shown in Figure 1 gave written informed consent, as outlined in the PLOS consent form, to publication of their photographs.

Because we use images of personally familiar faces in both Experiments 1 and 2, we require subject’s cooperation for identifying familiar individuals, making it impossible, or at best very difficult, to include images of unexpected familiar faces among the stimuli. Our results, therefore, are relevant for detection of faces when subjects are aware of the identities to be detected but not for detection of unexpected familiar identities.

Stimuli were presented with SuperLab (Cedrus, San Pedro, CA) with a viewing distance of 80 cm from the monitor (visual angle 6°).

The stimuli were presented in 1200 msec blocks of 15 face images each. Each face image was presented for 80 msec with no interval between stimuli. Each trial began with a fixation cross at the center of the screen for 1 s followed by a block of 15 face images and ended with a small square that indicated the time for the response.

#### Trials

There were seven trial types: 1–4) T1 followed by T2 after one to four fillers (stimulus onset asynchronies - SOAs - of 160 ms, 240 ms, 320 ms, and 400 ms), 5) T1 only, 6) T2 only, 7) catch trials (no targets). For each trial type with T2 stimuli (trial types 1–4 and 6) 30 trials had a personally familiar face T2 and 30 trials had an unfamiliar face T2. T1 was presented after the third to sixth filler stimulus. An interval of 2 sec followed each trial to allow the participants to respond.

Each subject saw 30 trials for each lag for the T1+T2 (familiar face) condition, 30 trials for each lag of the T1+T2 (unfamiliar face) condition, 30 trials for the T2 only (familiar face) condition, 30 trials for the T2 only (unfamiliar face) condition, 120 trials for the T1 only condition, and 120 catch trials (no T1 or T2). Images for individual faces were assigned randomly to each condition and lag.

#### Task

Participants were asked to indicate whether they detected human faces. Participants responded by pressing one of three keys on a keyboard: 1 when they saw only T1; 2 when they saw only T2; 3 when they saw T1+T2; and no response for catch trials. We chose to use inverted faces as T1 to ensure that subjects could distinguish T1 from T2 regardless of whether they could discern the identities of the faces.

A practice session preceded the experiment. Data from the practice session were not analyzed.

#### Statistics

We calculated detection rates for upright faces (T2) during the attentional blink using only trials on which the inverted face (T1) was detected. We analyzed T2 detection rates during the attentional blink using a two-way ANOVA with lag (4 SOAs) and familiarity (familiar versus unfamiliar) as factors.

### Results and Discussion

**Figure 2:**
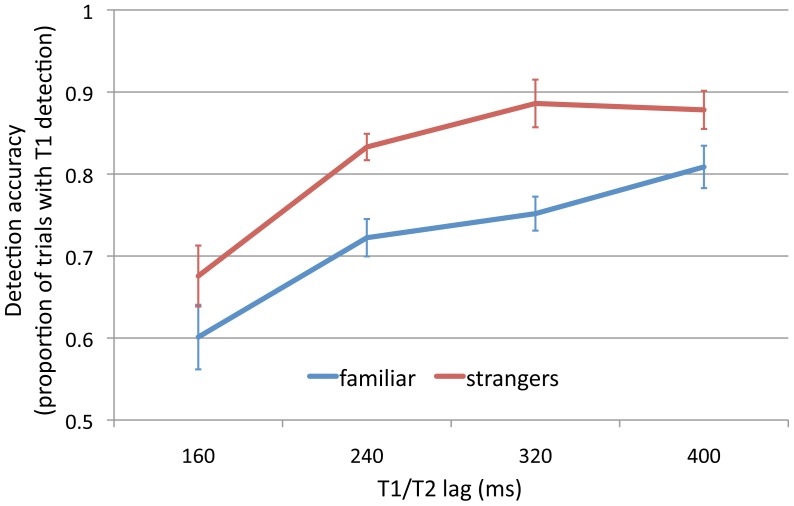
Results for each lag during the attentional blink. Personally familiar faces were detected more frequently than were faces of strangers during the attentional blink.

We found significant effects for familiarity (F_1, 84_ = 21.4, p<0.0001) and lag (F_3, 84_ = 18.9 p<0.0001, Figure 2). The interaction between familiarity and lag, however, was not significant (F_1, 84_ = 1.6, n.s. p>0.1).

Personally familiar faces were detected more frequently than were faces of strangers during the attentional blink, indicating that detection of familiar faces requires fewer attentional resources than does detection of unfamiliar faces.

These results suggest that detection of familiar faces is more robust during the attentional blink than is detection of unfamiliar faces and, thus, reflects a process that requires fewer attentional resources. The difference between familiar and unfamiliar face detection was significant for the intermediate SOAs (240 and 320 ms, t_12_ = 3.22 and 3.38, respectively, p<0.01 in both cases) and not significant for the shortest and longest lags. Detection rates for unfamiliar faces do not appear to have reached asymptote at an SOA of 400 ms, suggesting that the attentional blink was not fully resolved over the SOAs that we used.

A bias toward quick detection of personally familiar faces and stimuli that signify threat has been reported in the literature. In our experiment, we wanted to test if familiar faces are more resistant to the attentional blink than are faces of strangers. The most likely explanation for the attentional blink is a temporary engagement of attentional resources by the first target, reducing the resources that are available to process the second target [8], [9]. The attentional blink can be attenuated when the second target is an emotionally relevant stimulus such as a fearful face, as compared to a face with a neutral expression [11], [17], [18]. Faces with fearful expressions can modulate neural activity in brain areas that are involved in processing emotional stimuli even with reduced attentional resources and lack of awareness [1], [2], [4], [5], [19], [20]. These findings suggest the existence of mechanisms for quick recognition of stimuli that may signal threat. An alternative route that bypasses the visual ventral temporal pathway has been proposed for fast processing of this type of stimuli [21]. The existence of such a pathway in processing affective stimuli in humans has been questioned by others who suggest that the visual pathway may be sufficient for fast processing of these stimuli – even with reduced attentional resources and awareness – and, further, propose a role for top-down biasing of visual cortex involving frontal-parietal regions [22]–[24]. An alternative direct frontal-occipital pathway has been proposed to explain unconscious face processing in normal cognition and covert recognition in prosopagnosics [25].

The weaker susceptibility of familiar faces, as well as of threatening stimuli, to the attentional blink does not imply, of course, that similar mechanisms are used to process these stimuli, even though they are both socially relevant. For example, we have shown in a neuroimaging experiment that personally familiar faces induce a decrease in the amygdala response [26] unlike faces with fearful expression, which induce an increased response in this structure [27]. Our behavioral results cannot identify the neural systems involved during the attentional blink with personally familiar faces. Overlearned familiarity with the visual aspects of familiar faces, retrieval of person knowledge or both could play a fundamental role. Further experiments are needed to address this point.

Our results suggest that personally familiar faces are detected by mechanisms that require less attention than are required for unfamiliar faces, indicating that facilitated processing of socially meaningful stimuli can be learned through experience. To investigate further the extent to which familiar face detection is prioritized, we ran a second experiment to evaluate detection of familiar faces in condition of lack of awareness.

## Experiment 2 - Continuous Interocular Flash Suppression

### Methods

#### Subjects

20 volunteers (14 females; mean age: 23±4) participated in this experiment. Participants had no history of neurological or psychiatric diseases and had normal or corrected to normal vision. Participants gave written informed consent approved by the local ethical committee and were compensated for their participation.

#### Stimuli

**Figure 3:**
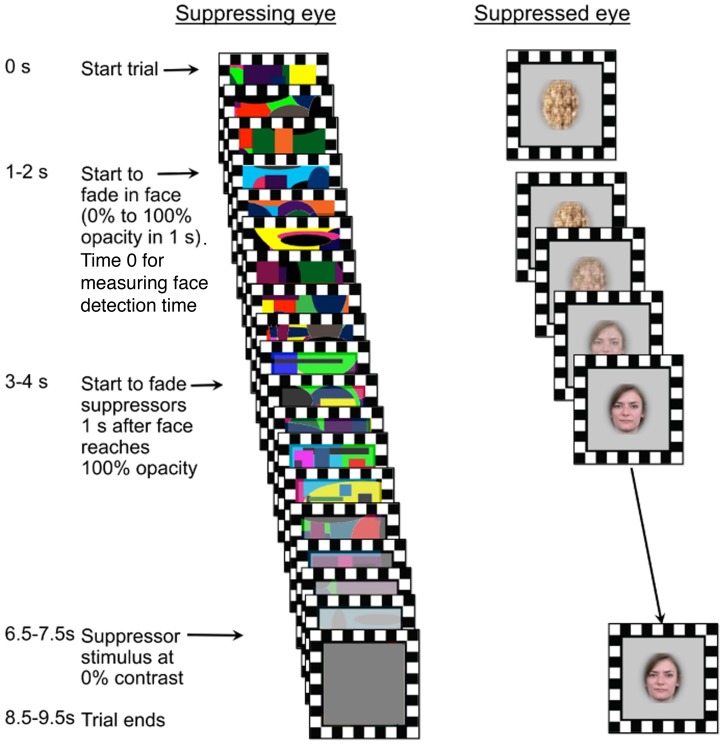
Continuous Flash Suppression (CSF) paradigm. Different high contrast collages of colored shapes were presented to one eye at 10 Hz. A phase-scrambled image that faded into an intact face image over 1 s was presented to the other eye. After the intact face was at full opacity for 1 s, the suppressing stimuli slowly faded to a gray square.

All participants were asked to provide contacts for 4 friends with whom they reported to have a good relationship and had known for more than a year. To ensure that all the stimuli were equal in terms of image quality, we made pictures of the friends of participants in a photo studio with identical lighting and camera placement and settings. To ensure that the unfamiliar control faces were truly strangers to the participants and that the pictures were of the same quality as the pictures of the familiar faces, we took pictures of students at another university (University of Vermont), where we set up a photo studio that was identical to the one at Dartmouth College. Pictures of strangers were matched in gender, age and race with the personally familiar faces. Six different face images of each of the four friends and four strangers were selected. Face images were in color, presented in an oval mask, subtending 1.6 degrees of horizontal visual angle and 2 degrees of vertical visual angle, on a gray surround. The subject shown in Figure 3 gave written informed consent, as outlined in the PLOS consent form, to publication of her photograph.

Suppressing stimuli were brightly colored, high contrast collages of different shapes (rectangular and curved figures), subtending 3 degrees of visual angle horizontally and vertically, that changed every 100 ms (Figure 3). The dynamic suppressing stimuli and the target stimuli were presented in central vision on different monitors with a mirror haploscope, mounted on a chin rest.

#### Trials

Each trial was preceded by 1s of a gray screen with a fixation cross. For the first 1 to 2 s of a trial, dynamic suppressing stimuli were presented at 10 Hz to one eye, and a phase-scrambled face image, with the same dimensions as the intact face images, was presented to the other eye (Figure 3). Phase-scrambled face images matched the intact faces in terms of spatial frequencies and luminance. The target face was faded in over 1 s by gradually increasing its opacity from 0% to 100%. Detection time was measured starting from the fading-in of faces. Beginning one second after the face image was at 100% opacity (2 to 3 s after trial onset), the contrast of the suppressing stimuli progressively decreased over 3.5 s to zero (a gray square). Each trial ended with presentation of the face image with no suppressing stimulus for 2s. For each subject, 96 trials had familiar faces and 96 trials had unfamiliar faces. We included 72 catch trials to prevent premature responses before true breakthrough. On catch trials, the phase-scrambled image was not replaced with an intact face image, and the trial ended with the phase-scrambled image and no suppressing stimulus.

#### Task

Subjects were instructed to respond by pressing the space bar on a keyboard as soon as they saw a face or any part of a face. Response times were measured relative to the time when a face began to fade in. Subjects were instructed to make no response on catch trials.

#### Statistics

We compared reaction times for detection of familiar faces as compared to faces of strangers. For each subject, we calculated the median reaction time for each of the two conditions. We used a matched-pair t-test (two-tailed) to assess the significance of the difference in time to break through interocular suppression for personally familiar faces as compared to faces of strangers.

### Results and Discussion

**Figure 4:**
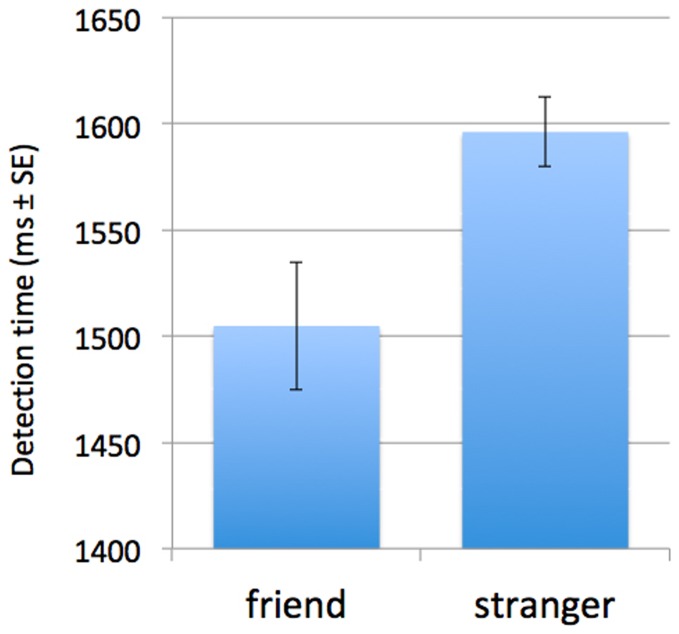
Results of the CSF experiment. Faces of friends were detected 91±40 SE ms faster than were faces of strangers.

We compared how long it took for faces of friends and faces of strangers to break through continuous flash interocular suppression. Faces of friends were detected 91±40 (SE) ms faster (1505 ms versus 1596 ms, t_19_ = 2.28, p<0.05, two-tailed) than were faces of strangers (Figure 4).

Continuous flash interocular suppression renders stimuli invisible using binocular rivalry with a high energy, rapidly changing stimulus presented to one eye [13]. If the stimuli to the two eyes are of equivalent salience, awareness of stimuli fluctuates spontaneously. With continuous flash interocular suppression it is possible to prevent one image to reach awareness for longer periods of time. We used continuous flash suppression so that onset of a new face image was not immediately visible, thus allowing processing of that image without awareness for an extended period of time.

Because a stimulus is subjectively invisible prior to breakthrough, any factor that facilitates faster breakthrough indicates processing that occurs without conscious awareness. Previous reports of faster breakthrough for some faces as compared to others have varied facial attributes that are presumably mediated by the more dorsal part of the distributed neural system for face perception [28]–[30], involving the superior temporal sulcus. These attributes have been fearful expression [5], [31], [32], head angle [7], and eye gaze [6]. By contrast, faster breakthrough based on identity is presumably mediated by the more ventral part of the distributed system for face perception, involving the fusiform gyrus and anterior temporal cortex [33], [34], with possible involvement of brain areas for social cognition, such as the medial prefrontal cortex and the temporoparietal junction [26], [29], [35]–[38]. The precise delineation of the neural systems that mediate facilitated processing of familiar faces requires further study, but it appears to be a different system than the system for facilitated processing of fearful faces.

## General Discussion

We conducted two experiments to investigate if personally familiar faces are processed in a prioritized way when attentional resources are reduced and when one is not consciously aware of the face image. In the first experiment we compared detection rate for personally familiar faces versus faces of strangers during the attentional blink. In the second experiment we measured how quickly personally familiar faces and faces of strangers break through interocular flash suppression. The results of the first experiment showed that familiar faces are more readily detected during the attentional blink than are faces of strangers. The results of second experiment showed a faster break through for personally familiar faces as compared to faces of strangers. Our findings indicate that faces that are personally familiar are processed preferentially in conditions of decreased attentional resources and lack of awareness.

Quick recognition of familiar people has an adaptive value and is essential for effective social interactions. A mechanism for recognition of familiar conspecifics is widespread in the animal kingdom. For example, monkeys are able to recognize the identity of other individual group members by their call and respond according to their social status [39]. In humans, recognition of a familiar individual is associated with the spontaneous retrieval of person knowledge about that individual [40]. Recognizing that a face is familiar, as compared to simply detecting a face, requires approximately an additional 100 ms of processing (according to Barragan-Jason et al.’s [41], [42] estimate). We have proposed that recognition of familiar individuals is the result of activation of a distributed neural system that involves not only the visual cortex but also areas that are implicated in nonvisual cognitive functions, such as the medial prefrontal cortex, the temporo-parietal junction, the precuneus, and the anterior temporal cortex – areas that play roles in Theory of Mind [43], [44] and autobiographical memory, and the amygdala and anterior insula – areas that play a role in emotional responses [26], [29], [35], [37], [40], [45], [46]. Activation of these systems may be due to the spontaneous retrieval of person knowledge and the emotional response that support successful recognition of familiar individuals. The higher detection rate for familiar faces during the attentional blink (Experiment 1) and processing of familiar faces without awareness (Experiment 2) suggest that part or all of the systems that mediate recognition of familiar individuals may be activated independently of explicit visual recognition. This hypothesis finds support also from the neuropsychological literature. Prosopagnosia is a neurological disorder characterized by the inability to explicitly recognize the identity of a familiar person based on the visual appearance of the face and in the absence of other cognitive impairments such as memory deficits or nonface object recognition [47]. Patients affected by this disorder, however, implicitly recognize familiar faces despite their inability to recognize them explicitly [48]–[50], as evidenced by normal augmentation of skin conductance response to familiar as compared to unfamiliar faces. Further behavioral evidence for implicit recognition of familiar faces comes from studies using a forced choice familiarity task, a forced choice cued task and a priming task [51]–[52]. The data from prosopagnosia indicate that detection of familiar individuals can happen without conscious visual recognition. Our data provide further support for the hypothesis that recognition of familiar individuals involves processes that do not require conscious awareness.

Signals that communicate threat also are processed at a preconscious level. Examples of such signals include faces with fearful expressions, vocal expressions of fear, bodily expressions of anger, spiders and snakes [4], [5], [10], [11], [53]. This capacity could be mediated by a separate pathway in the visual system for responding quickly to stimuli and events that might compromise our well-being [21]. A subcortical pathway through the superior colliculus and pulvinar has been hypothesized that mediates a coarser but faster processing of affective visual stimuli [20]. The existence of a subcortical pathway for quick detection of relevant stimuli has been questioned and alternatives that involve processing relevant stimuli through direct cortico-cortical connections have been proposed [25], [54]. Expressions of emotion such as fear activate the amygdala under conditions of increased attentional load [2] and in the absence of awareness [1]. The expression of fear alters the image of a face with a change in shape of the eyes, the eyebrows and the mouth. One of these features, exposure of the whites of the eyes, could be a simple, low spatial frequency feature that mediates rapid detection of fear without awareness [19]. More recent findings indicate that other social cues associated with faces, such as head angle or direct eye gaze, also are detected without awareness [6], [7]. Rapid detection of these facial cues could also be mediated by simple, low spatial frequency features, for example the central placement of the iris within the sclera or the central placement of the eyes and mouth in the oval of the face. In our experiment, we tightly controlled the quality of the stimuli. Pictures of familiar faces and control faces were matched on age, gender, and race, and were equated in terms of dimensions, light conditions and image quality. Therefore, our results cannot be ascribed to low-level feature differences between familiar and unfamiliar faces. Instead, the distinction between familiar and unfamiliar must be based on learned discrimination of facial configurations that are unique to individuals. Our results highlight that a socially-salient perceptual discrimination that is learned through experience is processed without awareness.

In conclusion the results of the present experiments provide evidence for preferential processing of stimuli that are socially salient and do not signal threat. Our results indicate that mechanisms for detection of socially-relevant stimuli with reduced attentional resources and even without conscious awareness can be due to learning of complex stimulus configurations.

## References

[1] Morris JS, Ohman A, Dolan RJ (1998) Conscious and unconscious emotional learning in the human amygdala. Nature 393: 467–470. 9624001 10.1038/30976

[2] Vuilleumier P, Armony JL, Driver J, Dolan RJ (2001) Effects of attention and emotion on face processing in the human brain: an event-related fMRI study. Neuron 30: 829–841. 11430815 10.1016/s0896-6273(01)00328-2

[3] Morton J, Johnson MH (1991) CONSPEC and CONLERN: A two-process theory of infant face recognition. Psychological Review 98: 164–181. 2047512 10.1037/0033-295x.98.2.164

[4] Jiang Y, Costello P, He S (2007) Processing of invisible stimuli: Advantage of upright faces and recognizable words in overcoming interocular suppression. Psychological Science 18: 349–355. 17470261 10.1111/j.1467-9280.2007.01902.x

[5] Zhou G, Zhang L, Liu J, Yang J, Qu Z (2010) Specificity of face processing without awareness. Consciousness and Cognition 19: 408–412. 20116293 10.1016/j.concog.2009.12.009

[6] Stein T, Shenju A, Peelen MV, Sterzer P (2011) Eye contact facilitates awareness of faces during interocular suppression. Cognition 119: 307–311. 21316650 10.1016/j.cognition.2011.01.008 PMC3796336

[7] Gobbini MI, Gors JD, Halchenko YO, Hughes HC, Cipolli C (2013) Processing of Invisible Social Cues. Consciousness and Cognition. In press. 10.1016/j.concog.2013.05.002 23727710

[8] Chun MM, Potter MC (1995) A two-stage model for multiple target detection in rapid serial visual presentation. J Exp Psychol Hum Percept Perform 21: 109–127. 7707027 10.1037//0096-1523.21.1.109

[9] Raymond JE, Shapiro KL, Arnell KM (1992) Temporary suppression of visual processing in an RSVP task: An attentional blink. Journal of Experimental Psychology: Human Perception and Performance 19: 849–860. 10.1037//0096-1523.18.3.849 1500880

[10] Anderson AK, Phelps EA (2001) Lesions of the human amygdala impair enhanced perception of emotionally salient events. Nature 411: 305–309. 11357132 10.1038/35077083

[11] Milders M, Sahraie A, Logan S, Donnellon N (2006) Awareness of faces is modulated by their emotional meaning. Emotion 6: 10–17. 16637746 10.1037/1528-3542.6.1.10

[12] Ramon M, Caharel S, Rossion B (2011) The speed of recognition of personally familiar faces. Perception 40: 437–449. 21805919 10.1068/p6794

[13] Tsuchiya N, Koch C (2005) Continuous flash suppression reduces negative afterimages. Nat Neurosci 8: 1096–1101. 15995700 10.1038/nn1500

[14] Tong F, Meng M, Blake R (2006) Neural bases of binocular rivalry. Trends Cogn Sci 10: 502–511. 16997612 10.1016/j.tics.2006.09.003

[15] Kang MS, Blake R (2011) An integrated framework of spatiotemporal dynamics of binocular rivalry. Front Hum Neurosci 5: 88. 21941473 10.3389/fnhum.2011.00088 PMC3171066

[16] Haxby JV, Ungerleider LG, Clark VP, Schouten JL, Hoffman EA, et al (1999) The effect of face inversion on activity in human neural systems for face and object perception. Neuron 22: 189–199. 10027301 10.1016/s0896-6273(00)80690-x

[17] Maratos FA, Moggs K, Bradley BP (2008) Identification of angry faces in the attentional blink. Cogn Emot 22: 1340–1352. 19360116 10.1080/02699930701774218 PMC2666369

[18] Martens S, Wyble B (2010) The attentional blink: past, present, and future of a blind spot in perceptual awareness. Neurosci Biobehav Rev 34: 947–957. 20025902 10.1016/j.neubiorev.2009.12.005 PMC2848898

[19] Whalen PJ, Kagan J, Cook RG, Davis FC, Kim H, et al (2004) Human amygdala responsivity to masked fearful eye whites. Science 306: 2061. 15604401 10.1126/science.1103617

[20] Tamietto M, de Gelder B (2010) Neural bases of the non-conscious perception of emotional signals. Nat Rev Neurosci 11: 697–709. 20811475 10.1038/nrn2889

[21] LeDoux J (2003) The Emotional Brain, Fear, and the Amygdala. Cellular and Molecular Neurobiology 23: 727–738. 14514027 10.1023/A:1025048802629 PMC11530156

[22] Dehaene S, Naccache L (2001) Towards a cognitive neuroscience of consciousness: basic evidence and a workspace framework. Cognition 79: 1–37. 11164022 10.1016/s0010-0277(00)00123-2

[23] Pessoa L, McKenna M, Gutierrez E, Ungerleider LG (2002) Neural processing of emotional faces requires attention. Proc Natl Acad Sci U S A 99: 11458–11463. 12177449 10.1073/pnas.172403899 PMC123278

[24] Rodríguez V, Thompson R, Stokes M, Brett M, Alvarez I, et al (2012) Absence of face-specific cortical activity in the complete absence of awareness: converging evidence from functional magnetic resonance imaging and event-related potentials. J Cogn Neurosci 24: 396–415. 21942763 10.1162/jocn_a_00137

[25] Valdés-Sosa M, Bobes MA, Quiñones I, Garcia L, Valdes-Hernandez PA, et al (2011) Covert face recognition without the fusiform-temporal pathways. Neuroimage 57: 1162–1176. 21570471 10.1016/j.neuroimage.2011.04.057

[26] Gobbini MI, Leibenluft E, Santiago N, Haxby JV (2004) Social and emotional attachment in the neural representation of faces. Neuroimage 22, 1628–1635. 10.1016/j.neuroimage.2004.03.049 15275919

[27] Whalen PJ, Rauch SL, Etcoff NL, McInerney SC, Lee MB, et al (1998) Masked presentations of emotional facial expressions modulate amygdala activity without explicit knowledge. J Neurosci 18: 411–418. 9412517 10.1523/JNEUROSCI.18-01-00411.1998 PMC6793390

[28] Haxby JV, Hoffman EA, Gobbini MI (2000) The distributed human neural system for face perception. Trends Cogn Sci 4: 223–233. 10827445 10.1016/s1364-6613(00)01482-0

[29] Gobbini MI, Haxby JV (2007) Neural systems for recognition of familiar faces. Neuropsychologia 45: 32–41. 16797608 10.1016/j.neuropsychologia.2006.04.015

[30] Haxby JV, Gobbini MI (2011) Distributed neural systems for face perception. In Calder AJ, Rhodes G, Johnson MH, Haxby JV (eds), Handbook of Face Perception. (Oxford: Oxford University Press).

[31] Bannerman RL, Milders M, De Gelder B, Sahraie A (2008) Influence of emotional facial expressions on binocular rivalry. Ophthalmic Physiol Opt 28: 317–326. 18565087 10.1111/j.1475-1313.2008.00568.x

[32] Yang E, Zald DH, Blake R (2007) Fearful expressions gain preferential access to awareness during continuous flash suppression. Emotion 7: 882–886. 18039058 10.1037/1528-3542.7.4.882 PMC4038625

[33] Kriegeskorte N, Formisano E, Sorger B, Goebel R (2007) Individual faces elicit distinct response patterns in human anterior temporal cortex. Proc Natl Acad Sci U S A 104: 20600–20605. 18077383 10.1073/pnas.0705654104 PMC2154477

[34] Rajimehr R, Young JC, Tootell RB (2009) An anterior temporal face patch in human cortex, predicted by macaque maps. Proc Natl Acad Sci U S A 106: 1995–2000. 19179278 10.1073/pnas.0807304106 PMC2632713

[35] Leibenluft E, Gobbini MI, Harrison T, Haxby JV (2004) Mothers’ neural activation in response to pictures of their, and other, children. Biol Psychiatry 56 225–232. 10.1016/j.biopsych.2004.05.017 15312809

[36] Gobbini MI, Koralek AC, Bryan RE, Montgomery KJ, Haxby JV (2007) Two takes on the social brain: a comparison of theory of mind tasks. J Cogn Neurosci 19: 1803–1814. 17958483 10.1162/jocn.2007.19.11.1803

[37] Taylor MJ, Arsalidou M, Bayless SJ, Morris D, Evans JW, et al (2009) Neural correlates of personally familiar faces: parents, partner and own faces. Hum Brain Mapp 30: 2008–2020. 18726910 10.1002/hbm.20646 PMC6870744

[38] Gobbini MI, Gentili C, Ricciardi E, Bellucci C, Salvini P, et al (2011) Distinct neural systems involved in agency and animacy detection. J Cogn Neurosci 23: 1911–1920. 20849234 10.1162/jocn.2010.21574

[39] Cheney DL, Seyfarth RM (1999) Recognition of other individuals’ social relationships by female baboons. Anim Behav 58: 67–75. 10413542 10.1006/anbe.1999.1131

[40] Gobbini MI (2010) Distributed process for retrieval of person knowledge. In Todorov A, Fiske ST, & Prentice D (Eds), Social neuroscience: Toward understanding the underpinnings of the social mind (pp. 40–53). New York: Oxford University Press.

[41] Barragan-Jason G, Lachat F, Barbeau EJ (2012) How Fast is Famous Face Recognition? Front Psychol 3: 454. 23162503 10.3389/fpsyg.2012.00454 PMC3498873

[42] Barragan-Jason G, Besson G, Ceccaldi M, Barbeau EJ (2013) Fast and Famous: Looking for the Fastest Speed at Which a Face Can be Recognized. Front Psychol 4: 100. 23460051 10.3389/fpsyg.2013.00100 PMC3586696

[43] Frith CD, Frith U (2006) The neural basis of mentalizing. Neuron 50: 531–534. 16701204 10.1016/j.neuron.2006.05.001

[44] Saxe R, Powell LJ (2006) It’s the thought that counts: specific brain regions for one component of theory of mind. Psychol Sci 17: 692–699. 16913952 10.1111/j.1467-9280.2006.01768.x

[45] Bartels A, Zeki S (2000) The neural basis of romantic love. Neuroreport 11: 3829–3834. 11117499 10.1097/00001756-200011270-00046

[46] Gobbini MI, Haxby JV (2006) Neural response to the visual familiarity of faces. Brain Research Bulletin 71: 76–82. 17113931 10.1016/j.brainresbull.2006.08.003

[47] Damasio AR, Damasio H, Van Hoesen GW (1982) Prosopagnosia: anatomic basis and behavioral mechanisms. Neurology 32: 331–341. 7199655 10.1212/wnl.32.4.331

[48] De Haan EH, Bauer RM, Greve KW (1992) Behavioural and physiological evidence for covert face recognition in a prosopagnosic patient. Cortex 28(1): 77–95. 1572175 10.1016/s0010-9452(13)80167-0

[49] Schweinberger SR, Burton AM (2003) Covert recognition and the neural system for face processing. Cortex 39: 9–30. 12627750 10.1016/s0010-9452(08)70071-6

[50] Tranel D, Damasio AR (1985) Knowledge without awareness: an autonomic index of facial recognition by prosopagnosics. Science 228, 1453–1454. 10.1126/science.4012303 4012303

[51] Avidan G, Behrmann M (2008) Implicit familiarity processing in congenital prosopagnosia. J Neuropsychol 2: 141–64. 19334309 10.1348/174866407x260180

[52] Rivolta D, Schmalzl L, Coltheart M, Palermo R (2010) Semantic information can facilitate covert face recognition in congenital prosopagnosia. J Clin Exp Neuropsychol 32: 1002–1016. 20437285 10.1080/13803391003662710

[53] Alpers GW, Gerdes ABM, Lagarie B, Tabbert K, Vaitl D, et al (2009) Attention and amygdala activity: an fMRI study with spider pictures in spider phobia. J. Neural Transm 116: 747–757. 18726545 10.1007/s00702-008-0106-8

[54] Pessoa L, Adolphs R (2010) Emotion processing and the amygdala: from a ‘low road’ to ‘many roads’ of evaluating biological significance. Nat Rev Neurosci 11: 773–783. 20959860 10.1038/nrn2920 PMC3025529

